# Bilateral striatal necrosis due to homoplasmic mitochondrial 3697G>A mutation presents with incomplete penetrance and sex bias

**DOI:** 10.1002/mgg3.541

**Published:** 2019-01-08

**Authors:** Shanshan Zhong, Shumeng Wen, Yusen Qiu, Yanyan Yu, Ling Xin, Yang He, Xuguang Gao, Hezhi Fang, Daojun Hong, Jun Zhang

**Affiliations:** ^1^ Department of Neurology Peking University People’s Hospital Beijing China; ^2^ Key Laboratory of Laboratory Medicine, College of Laboratory Medicine and Life Science Wenzhou Medical University Wenzhou China; ^3^ Department of Neurology The First Affiliated Hospital of Nanchang University Nanchang China; ^4^ Department of Health, Exercise Science, and Recreation Management University of Mississippi University Mississippi

**Keywords:** bilateral striatal necrosis, homoplasmy, mitochondrial DNA mutation, sex bias

## Abstract

**Background:**

Heteroplasmic mitochondrial 3697G>A mutation has been associated with leber hereditary optic neuropathy (LHON), mitochondrial encephalopathy, lactic acidosis and stroke‐like episodes (MELAS), and LHON/MELAS overlap syndrome. However, homoplasmic m.3697G>A mutation was only found in a family with Leigh syndrome, and the phenotype and pathogenicity of this homoplasmic mutation still need to be investigated in new patients.

**Methods:**

The clinical interviews were conducted in 12 individuals from a multiple‐generation inherited family. Mutations were screened through exome next‐generation sequencing and subsequently confirmed by PCR‐restriction fragment length polymorphism. Mitochondrial complex activities and ATP production rate were measured by biochemical analysis.

**Results:**

The male offspring with bilateral striatal necrosis (BSN) were characterized by severe spastic dystonia and complete penetrance, while the female offspring presented with mild symptom and low penetrance. All offspring carried homoplasmic mutation of NC_012920.1: m.3697G>A, p.(Gly131Ser). Biochemical analysis revealed an isolated defect of complex I, but the magnitude of the defect was higher in the male patients than that in the female ones. The ATP production rate also exhibited a similar pattern. However, no possible modifier genes on the X chromosome were identified.

**Conclusion:**

Homoplasmic m.3697G>A mutation could be associated with BSN, which expanded the clinical spectrum of m.3697G>A. Our preliminary investigations had not found the underlying modifiers to support the double hit hypothesis, while the high level of estrogens in the female patients might exert a potential compensatory effect on mutant cell metabolism.

## 1 INTRODUCTION

Bilateral striatal necrosis (BSN) is a heterogeneous group of neurodegenerative diseases named after the distinctive neuroradiological features (Lim, 2009). The main neurological findings are characterized by symmetrical degeneration of putamen, caudate nucleus, and globus pallidus occasionally. The pathogenesis of BSN is heterogeneous and has been associated with various acquired and hereditary causes (Chokshi, Aygun, & Mullins, 2014). Hereditary striatal degeneration can be inherited as an autosomal dominant, autosomal recessive, or mitochondrial disorder (Tonduti et al., 2016). Adult‐onset autosomal‐dominant BSN is caused by mutations in the phosphodiesterase 8B (*PDE8B*, OMIM #603390) gene on chromosome 5q13 (Appenzeller et al., 2010). Autosomal‐recessive BSNs are associated with mutations in several genes such as solute carrier family 25 member 19 (*SLC25A19*, OMIM #606521) (Spiegel et al., 2009), nucleoporin 62 (*NUP62*, OMIM #605815) (Bianciardi et al., 2016), adenosine deaminase RNA specific (*ADAR1*, OMIM: 146920) (La Piana et al., 2014), and protein Vac14 homolog (*VAC14*, OMIM #604632) (Stutterd et al., 2017). Mitochondrial BSNs are caused by mutations in both mitochondrial and nuclear genes associated with the maintenance of mitochondrial function (Binder et al., 2003; Thyagarajan, Shanske, Vazquez‐Memije, Vivo, & DiMauro, 1995).

Several mitochondrial DNA (mtDNA) mutations associated with defects of oxidative respiratory chain enzymes were linked with infantile‐onset BSNs (Hirayanagi et al., 2017; Tarnopolsky, Meaney, Robinson, Sheldon, & Boles, 2013). The heteroplasmic mutation of NC_012920.1: m.3697G>A, p.(Gly131Ser) in the mitochondrial NADH dehydrogenase subunit 1 (*MTND1*, OMIM #516000) gene was identified in patients with mitochondrial encephalomyopathy, lactic acidosis and stroke‐like episodes (MELAS) (Kirby et al., 2004), Leber's hereditary optic neuropathy (LHON) (Spruijt et al., 2007), LHON/MELAS overlap syndrome (Blakely et al., 2005), and Stüve‐Wiedemann syndrome (Morava, Hamel, Hol, Rodenburg, & Smeitink, 2006). In contrast, the homoplasmic m.3697G>A mutation was found as a causation of Leigh syndrome (LS) in a Japanese family (Negishi et al., 2014), in which the affected individuals exhibited early childhood‐onset rigidity of the upper and lower limbs due to bilateral basal ganglion and brainstem degeneration. Herein, we investigated the clinical features, brain MRI changes, genetic findings, and mitochondrial function in a family with BSN and assessed its association with homoplasmic m.3697G>A mutation.

## 2 MATERIALS AND METHODS

### 2.1 Ethical compliance

The clinical examinations and genetic analyses in this family were conducted after the written informed consent was provided by every examined individual according to the Declaration of Helsinki. The study was approved by the institutional ethical review board of the Peking University People's Hospital.

### 2.2 Patients

**Figure 1:**
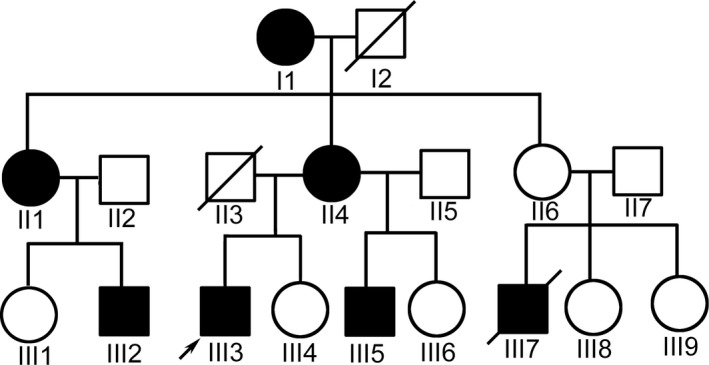
The pedigree of family: squares, male; circles, female; black symbols, affected; arrow, the index

The index patient was a 20‐year‐old man (III3) from a nonconsanguineous family (Figure 1). He had rigidity on the left limbs since he was 6 years old. The dystonia insidiously progressed and resulted in clumsy movements of the left limbs, but the symptoms maintained a stable condition since the age of eight. Eight months ago, the right limbs started exhibiting similar rigidity. He presented with dysarthria, dysphagia, and difficulty of standing steadily. The patient had no complaints about muscle weakness, myalgia, or numbness. Mini‐mental state examination (MMSE) revealed a moderate cognitive disability (17/30). Physical examination revealed scoliosis, finger joint contracture, left ankle deformity, and an increased muscle tone of the neck and all of the limbs. Muscle strength graded 5/5 in the right limbs and 4/5 in the left limbs (Medical Research Council Scale). The deep tendon reflexes of the lower limbs were enhanced. The Babinski sign was positive on the left side. His visual acuity and auditory ability were unimpaired. The pinprick, position, and vibration sensations were normal.

The elder aunt's son (III2) was 23 years old, and he displayed spastic gait at the age of five. He developed upper limb spasticity, lower limb tightness, and walking difficulty at the age of 10. He was wheelchair‐bound when he was referred to our clinic at the age of 26. His cognitive performance was mildly decreased (MMSE: 20/30). Physical examination revealed enhanced muscle tone. The sensations were intact, and the optic neuropathy was not detected.

The index patient's young brother (III5) was 16 years old. He had similar symptoms with the index patient. The age of onset was four. Clinical symptoms included moderate cognitive ability (MMSE: 17/30), scoliosis, finger joint contracture, deformity of the ankle joint, and enhanced muscle tone. No sensations or optic nerves were impaired.

The mother (II4) was 47 years old and started having slight clumsiness of left upper limbs at the age of 18, but the symptoms had been stable since she was 20 years old. Physical examination revealed an enhanced muscle tone on the left upper limbs. No other abnormalities were detected.

The elder aunt (II1) was reported to have clumsy movement of right limbs since 17 years old, and the symptoms had not progressed since she was 20 years old. The grandmother (I1) had rigid hands in her 20 s, but quickly became progression‐free. The young aunt (II6) had no complaints of any symptoms, but her son (III7) had a similar clumsy gait as that of the index patient and died of severe injury at age 17. The detailed data were summarized in Table 1. The family individuals (III1, III4, III6, III8, and III9) were not found to have any symptoms or abnormal signs.

**Table 1:** The clinical features and the laboratory findings in the BSN family with homoplasmic m.3697G>A

Patient	Age/gender	Age at onset	Clinical features	Optic symptoms	MMSE	Cerebral MRI	Mutation
I1	73/F	20	Hands rigidity	None	24	NA	NA
II1	50/F	17	Right limbs clumsy	None	30	Left hyperintensities of the putamen	Homoplasmic m.3697G>A
II4	47/F	18	Left limb rigidity	None	29	Right hyperintensities of the putamen	Homoplasmic m.3697G>A
II6	44/F	NA	None	None	30	Normal	Homoplasmic m.3697G>A
III1	25/F	NA	None	None	30	Normal	Homoplasmic m.3697G>A
III2	23/M	5	Spastic dystonia	None	20	Bilateral hyperintensities of the putamen	Homoplasmic m.3697G>A
III3	20/M	6	Spastic dystonia	None	17	Bilateral hyperintensities of the putamen	Homoplasmic m.3697G>A
III4	18/F	NA	None	None	30	Normal	Homoplasmic m.3697G>A
III5	16/M	4	Spastic dystonia	None	17	Bilateral hyperintensities of the putamen and caudate nucleus	Homoplasmic m.3697G>A
III6	9/F	NA	None	None	27	Normal	Homoplasmic m.3697G>A
III7	17^a^/M	5	Spastic dystonia	NA	NA	Bilateral hyperintensities of the putamen	NA

Notes

F: female; M: male; MMSE: mini‐mental state examination; NA: unavailable.

a The patient died at the age of 17.

### 2.3 Brain MRI

Brain MRI was conducted by a 3.0‐T MR scanner (Seimen). Conventional T1‐weighted spin echo (T1WI) sequences were obtained with the following parameters: repetition time (TR) = 250 ms, echo time (TE) = 2.5 ms, FoV = 306 × 220 mm; conventional T2‐weighted spin echo (T2WI) sequences: TR = 4,000 ms, TE = 157 ms, FoV = 306 × 220 mm; fluid‐attenuated inversion recovery (FLAIR) sequences: TR = 8,000 mm, TE = 79 mm, FoV = 306 × 220 mm; diffusion tensor imaging (DTI): TR = 7,700 mm, TE = 104 mm, FoV = 315 × 227 mm; magnetic resonance spectroscopy (MRS): TR = 1,900 mm, TE = 2.3 mm, FoV = 695 × 500 mm. The slice thickness was 5 mm, and the slice gap was 6.5 mm.

### 2.4 Mutation analysis

Genomic DNA was extracted from peripheral blood samples of family members II4, III3, and III4. Initially, the genetic test was conducted through exome next‐generation sequencing (NGS). Targeted exon enrichment was performed using SureSelect Human All Exon V4 (Agilent Technologies, Santa Clara, CA, USA). The exon‐enriched DNA libraries were subjected to paired‐end sequencing with the Hiseq2000 platform (Illumina, Inc., San Diego, CA, USA). Calls with variant quality <20 were filtered out, and 95% of the targeted bases were covered sufficiently to pass our thresholds for calling single‐nucleotide polymorphisms (SNP) and small insertions or deletions.

Whole mtDNA sequencing was conducted after long‐PCR amplification. Amplified fragment was directly sequenced using a BigDye ready reaction kit (Applied Biosystems, Foster City, CA, USA) and was run on an ABI 3700 automated sequencer (Applied Biosystems). Identical mtDNA fragment from the family members (II1, II4, II6, III1, III2, III4, III5, and III6) was examined for the presence of the mutation.

In order to detect the mutant load of m.3697G>A, NGS targeted to MitoExome was performed using the blood and urine sediments of the index patient (III3) and the blood of member II4, III5, III6 (PrecisionMD Technology, Beijing, China). The average depth of reads for single nucleotide reached over 5,000 times, enabling the detection of heteroplasmic variants at levels of <0.1%. In addition, mutant load of m.3697G>A was further assessed by PCR‐restriction fragment length polymorphism (PCR‐RFLP). The mtDNA fragments were initially amplified using the primers as follows: forward 5′‐TACTTCACAAAGCGCCTTCC‐3′ and reverse 5′‐ATGAAGAATAGGGCGAAGGG‐3′, then cleaved by BclI restriction endonuclease (NEB, Ipswich, MA, USA), and finally separated by electrophoresis. The intensity of the bands was quantified by Gel‐Pro Analyzer 4.0 (MediaCybernetics, Warrendale, PA, USA). GenBank NC_012920.1 was adopted as the reference sequence.

The mtDNA sequence of the index patient carrying the m.3697G>A mutation was initially analyzed through MitoTool software (www.mitotool.org/) and then assigned to the Asian mitochondrial haplogroups by using the nomenclature of mitochondrial haplogroups (Tanaka et al., 2004).

### 2.5 Biochemical analysis

B‐lymphocytes from several family individuals (II4, III3, III5, and III6) were immortalized as described previously (Hammerschmidt & Sugden, 1989). Individual oxidative phosphorylation system (OXPHOS) complexes were extracted from mitochondria using *n*‐dodecyl‐β‐d‐maltoside (Sigma, St Louis, MO, USA) with a detergent/protein ratio of 2.5 g/g. Proteins (60 μg) containing 0.5% Blue G‐250 (Sigma) and 5% glycerol were separated via electrophoresis on 3%–11% gradient blue native polyacrylamide gels as previously described (Xu et al., 2017).

ATP production rate was assessed using an ATP measurement kit (Molecular Probes, Carlsbad, CA, USA) according to the manufacturer's instructions. To measure mitochondrial ATP level, cells were incubated with 10 mM glucose (Sigma) or 5 mM 2‐DG with 5 mM pyruvate for 2 hr prior to the measurement. Fluorescence/luminescence was measured using a Varioskan^TM^ Flash Multimode Reader (Thermo Fisher Scientific, Waltham, MA, USA).

## 3 RESULTS

### 3.1 Brain MRI features

**Figure 2:**
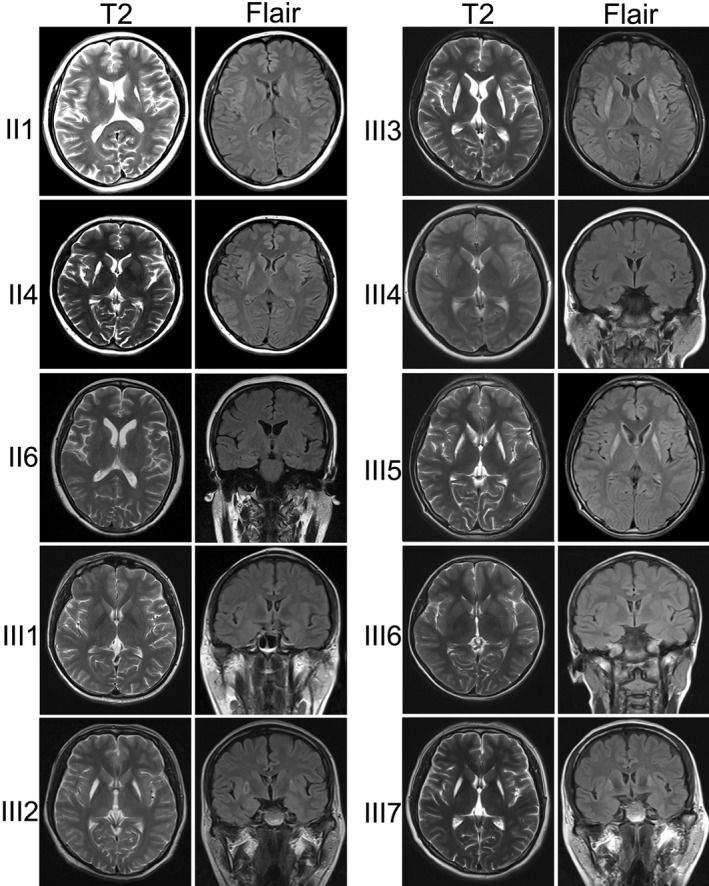
Brain MRI findings of family individuals. Magnetic resonance images of patients showed high‐intensity signals in the bilateral putaminal areas on both T2‐weighted (T2) and fluid‐attenuated inversion recovery (FLAIR) images in male patients III2, III3, III5, and III7; unilateral putaminal areas in female patients II1 and II4; and normal cerebral structure in other female individuals II6, III1, III4, and III6

Brain MRI of the index patient displayed high signal intensities in the bilateral putamen on T2WI and mildly high signal intensities on FLAIR images (Figure 2; III3). Abnormal signals in the bilateral putamen were also observed in the images of all other affected male individuals (Figure 2; III2, III5, and III7). In addition, high‐intensity signals in the bilateral caudate nucleus were observed in the images of patient III5 (Figure 2; III5). The elder aunt (II1) only had a high‐intensity signal in the left putamen (Figure 2; II1), and the mother (II4) had high‐intensity signal only in the right putamen (Figure 2; II4). In contrast to the male individuals, the female patient II6 (at age 45), patient III1 (at age 29), patient III4 (at age 18), and patient III6 (at age 9) had normal brain MRI images. The signal intensities were negatively related to enhancement in the lesions, but the lesions showed the fiber interruption on DTI images and severe decrease of *n*‐acetylaspartate (NAA) on MRS measure (Supporting Figure S1).

### 3.2 Mitochondrial DNA mutation

**Figure 3:**
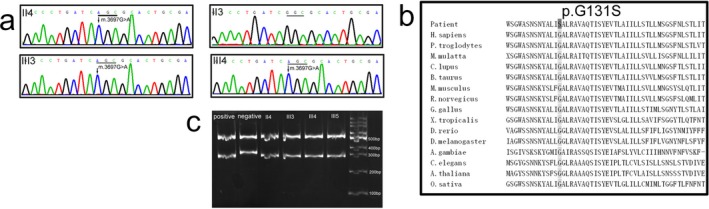
The homoplasmic mutation of NC_012920.1: m.3697G>A, p.(Gly131Ser) was found in the index patient (III3), his mother (II4), and his sister (III4), but not his father (II3) (a). The mutation changes a very evolutionarily conserved glycine into serine at 131 amino acid of MTND1 (b). The homoplasmy of m.3697G>A mutation was confirmed by PCR‐RFLP. The wild‐type fragment will be cleaved into two fragments of 532 bp and 300 bp, while the mutant fragment will be cut into three fragments of 532 bp, 270 bp, and 30 bp (c)

The homoplasmic mutation of NC_012920.1: m.3697G>A, p.(Gly131Ser) was detected in the index patient (III3) through mtDNA Sanger sequence (Figure 3a), which changes a very evolutionarily conserved glycine into serine at 131 amino acid of MTND1 (p.Gly131Ser; Figure 3b). The same homoplasmic mutation was identified in the circulating lymphocytes from the available family individuals (II1, II3, II4, II6, III1, III2, III4, III5, and III6). Targeted NGS for MitoExome showed that the index patient had more than 99.9% of m.3697G>A mutation in the circulating lymphocytes, and more than 99.9% of m.3697G>A mutation in the urinary epithelium; the mother (II4) had more than 99.9% of m.3697G>A mutation in the circulating lymphocytes; and the asymptomatic sister (III4) had more than 99.9% of m.3697G>A mutation in the circulating lymphocytes (Table 2).

**Table 2:** The targeted next‐generation sequencing for mitochondrial DNA in the index patient (III3), his mother (II4), and his younger sister (III4)

Sample	Read G (wt)	Read A	Read C	Read T	Heteroplasmy (%)
Patient					
Blood	0	6,016	1	1	99.97
Urine	1	5,381	0	1	99.96
Mother blood	0	5,958	0	1	99.98
Sister blood	1	5,839	1	0	99.97

Notes

Data are the number of reads for the specific nucleotide (A, C, G, T) with G as the wild type (wt) and A as the mutant. The percentage of heteroplasmy is the ratio of mutant/wt. The very low proportion of G, C, and T reads likely represents nonspecific sequence “noise.” The true percentage of heteroplasmy is more than 99.9% and likely represents homoplasmy for the mutant.

In the PCR‐RFLP analysis, the wild‐type *MTND1* fragment would be cleaved into two small fragments of 532 bp and 300 bp, while the mutant *MTND1* fragment would be cut into three small fragments of 532 bp, 270 bp, and 30 bp. The PCR‐RFLP showed that no 300 bp fragment was detected in the samples from patient II4, III3, III4, and III5, which indicated that the mutant loads of m.3697G>A were 100% in these patients (Figure 3c).

From the whole mtDNA sequencing of the blood from the index patient, the following polymorphisms were found: m.709T>C, m.3759A>G, m.5153A>G, m.5178C>T, m.9180A>G, m.10397A>G, m.15724A>G, m.16189T>C, and m.16519T>C. The results indicated that this family belonged to the mitochondrial haplogroup D5b1b, which was commonly found in Japan and North China. This haplogroup had been reported to be associated with schizophrenia, Parkinson disease, and diabetes in Japanese population, but had not been linked to BSN, LS, or LHON. Except for the homoplasmic mutation m.3028A>T, all the other polymorphisms were known as variations of the Cambridge sequence (Supporting Information Table S1).

### 3.3 Biochemical results

**Figure 4:**
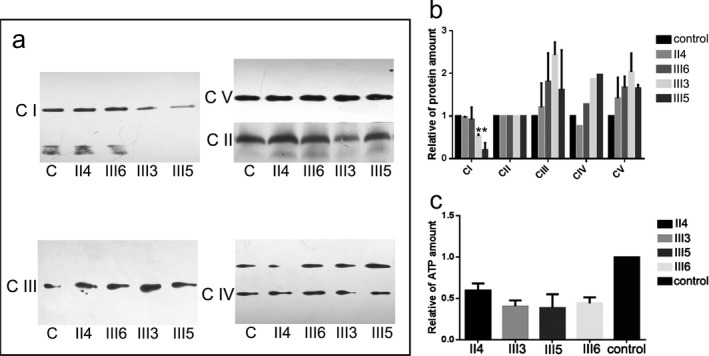
Blue Native PAGE blot revealed an isolated decrease of OXPHOS complex I in the male patients III3 and III5 compared with that of the female patients II4, III6, and a control; the other OXPHOS complexes were not impaired (a,b). The ATP production rate was significantly decreased in the male patients III3 and III5 compared with that of the female patients II4, III6, and a control (c). C: control; CI: complex I; CII: complex II; CIII: complex III; CIV: complex IV; CV: complex V

To investigate the pathogenic role of m.3697G>A and the possible cause of the sex bias, the levels of OXPHOS complexes were measured in immortalized lymphocytes derived from several family individuals. Blue Native PAGE/Immunoblot analysis revealed a significantly decreased level of OXPHOS complex I in the lymphocytes of the male patients III3 and III5 compared with that of the female patients II4, III6, and a control, but the other OXPHOS complexes were not impaired (Figure 4a,b). Consistently, the ATP production rate was significantly decreased in the male patient III3 and III5 compared with that of the female patient II4, III6, and a control (Figure 4c).

### 3.4 Genomic DNA mutation

Initially, the exome NGS did not find variants in the *PDE8B* gene for autosomal‐dominant striatal degeneration, or in the *SLC25A19*, *NUP62*, *ADAR1*, and *VAC14 *genes for autosomal‐recessive BSN. In addition, no variants were found in the NADH:Ubiquinone oxidoreductase subunit A1 (*NDUFA1*, OMIM #300078) gene or in the NADH:Ubiquinone oxidoreductase subunit B11 (*NDUFB11*, OMIM #300403) gene, both of which were located on the X chromosome. Owing to the obvious female bias in the family, we carefully compared the variants on the X chromosome between the index patient (III3) and his sister (III4) in the exome database. The filtering strategy was followed as a hemizygote in the index patient and a heterozygote in his sister on the X chromosome. We identified two candidate genes (Supporting Information Table S2). The shroom family member 2 (*SHROOM2*, OMIM #300103) gene showed a missense mutation NC_000023.11:c.1426G>C, p.(Gly476Arg) with a low allele frequency of 0.001796 in the gnomAD database. The leucine zipper protein 4 (*LUZP4*, OMIM #300616) gene had a missense mutation NM_016383.4:c.382A>G, p.(Asn128Asp) with a low allele frequency 0.0002301 in the gnomAD database.

## 4 DISCUSSION

The heteroplasmic m.3697G>A mutation had been associated with MELAS (Kirby et al., 2004), LHON (Spruijt et al., 2007), LHON/MELAS overlap syndrome (Blakely et al., 2005), and synergetic effects for Stuve‐Wiedemann syndrome (Morava et al., 2006). In contrast, the homoplasmic m.3697G>A mutation was only reported in a family with LS (Negishi et al., 2014). By reviewing the literatures about m.3697G>A mutation (Supporting Information Table S3), we found that the patient 1 reported by Negishi and colleagues indeed presented with a phenotype of early child‐onset BSN and relatively good prognosis (Negishi et al., 2014), and the younger brother described by Spruijt and colleagues should be patient with early child‐onset BSN and a stationary condition (Spruijt et al., 2007). Our patients also presented with child‐onset spastic dystonia selectively involved in the bilateral putaminal lesions. At least eight other variants of mtDNA could cause BSN that was characterized by spastic dystonia (Campos et al., 1997; Solano et al., 2003). Among those variants, the phenotype of m.14459G>A mutation showed a great similarity with that of m.3697G>A. The patients with m.14459G>A mutation also presented with LHON, dystonia, and LS/Leigh‐like syndrome (LLS) (Kim, Ki, & Park, 2010; Ronchi et al., 2011). Indeed, optic atrophy, childhood‐onset dystonia, BSN, LLS, and LS can be considered as a phenotypic spectrum of severity likely determined by the mutational heteroplasmy (Gropman et al., 2004).

The nomenclature of BSN is based on neuroradiological alterations, especially the degeneration of bilateral putamen, globus pallidus, and caudate nucleus. Our male patients showed bilateral putamen degeneration, except for the patient III5 who had an additional involvement of bilateral caudate nucleus. However, the affected female patients only presented with unilateral putamen degeneration. The asymmetrical lesions were also observed in the LS patient with homoplasmic m.3697G>A mutation (Negishi et al., 2014). This phenomenon suggested that the basal ganglion lesions had great heterogeneity in patients with homoplasmic m.3697G>A mutation. The typical phenotype of LS is a subacute progressive necrotizing encephalomyelopathy with poor prognosis characterized by bilateral symmetric necrosis of gray matter nuclei in the basal ganglia, diencephalons, cerebellum, or brainstem (Rahman et al., 1996). The radiological features of BSN overlapped greatly with, but not identical to those of LS. Therefore, the m.3697G>A mutation‐associated BSN, particularly the one confined to the putamen, might be considered as an abortive pattern of LS.

The disease severity and penetrance of mitochondrial diseases have been considered the results of mutation loads. For example, cases with m.8993T>G homoplasmy at a high mutation load caused LS, whereas lower mutation load resulted in a less severe phenotype (Sofou et al., 2018). Similarly, heteroplasmy of m.3697G>A might cause various mitochondrial disorders that presumably depended on the level of heteroplasmy. To our knowledge, homoplasmic mutation was only reported to be associated with LS (Negishi et al., 2014). In the current study, the individuals carrying homoplasmic m.3697G>A mutation presented with highly variable phenotypes, especially more severe clinical severity in the male patients than the females. The biochemical analysis revealed that the mutation caused an isolated defect of complex I, but the defect magnitude was higher in male patients than female ones, which can be the possible reason for the most striking discrepancy of clinical phenotype between the male and female patients. However, the underlying causes for the sex bias should be further explored.

Patients with different ethnic groups likely have different mtDNA haplogroups, although a precise definition of the haplotypes is available in few subjects (Qu et al., 2010). In this family, there were 45 variants in the mitochondrial genome, which pointed to the haplogroup D5b1b. This haplogroup had been reported to be associated with schizophrenia, Parkinson disease, and diabetes in Japanese population (Tanaka et al., 2004). Although mitochondrial haplogroups could influence the phenotypic presentation of BSN and LS, no evidence could be found between m.3697G>A and haplotype D5b1b disclosed in our patients.

A double hit hypothesis of disease predicts that only those who also inherit an X‐linked recessive phenotype modifier will actually develop the mitochondrial disease (Chen, Chen, & Guan, 2015). Apparently, the sex bias in this BSN family suggested that one or more modifier genes on the X chromosome might be responsible for the phenotypic variability and incomplete penetrance. By exome NGS screening, we did not find any variants in the *NDUFA1* and *NDUFB11 *genes that encode the protein subunits of complex I on the X chromosome. We found two suspicious genes (*Shroom2* and *LUZP4*) that closely co‐segregated with the phenotype in the family. Shroom2 is implicated in amiloride‐sensitive sodium channel activity that may be involved in endothelial cell morphology changes during cell spreading. Depletion of this gene results in an increase in endothelial sprouting, migration, and angiogenesis. This gene is highly expressed in the retina and may regulate the biogenesis of melanosomes and promote their association with the apical cell surface by inducing gamma‐tubulin redistribution, but it is supposed not to be expressed in the brain tissue (Farber, Rizaldy, & Hildebrand, 2011). LUZP4 is an export adapter that enhances the RNA‐binding activity of the nuclear RNA export factor NXF1 and can restore mRNA export function in cells compromised by loss of mRNA export adapters. It is highly expressed in cancer cells, but reported not to be expressed in the brain tissue (Viphakone et al., 2015). Therefore, the above genes were less likely to take synergetic effects on BSN, though they should be further investigated.

Significant sex bias had been universally described in the LHON pedigrees, in which the male offspring presented with more severe visual loss and higher penetrance, but the female offspring exhibited mild symptoms and low penetrance (Hudson et al., 2005). Similarly, no definite genetic modifiers were identified in these LHON patients. However, studies on LHON have revealed that estrogens could exert a potential compensatory effect on mutant cell metabolism through lowering oxidative stress and thus would reduce damage to the complex I proteins and increase ATP synthesis (Giordano et al., 2011). Future studies should be warranted to explore, yet it is uncertain whether estrogens can exert a potential effect on the phenotype of BSN.

In summary, this study identified homoplasmic m.3697G>A mutation as a cause of BSN and supported the role of MTND1 as a key player in the complex I. Although the female ancestors transmitted the BSN‐associated mtDNA mutation to all of their offspring, most females never presented with clinical symptoms and exhibited variably reduced penetrance with a sex bias that favored in the males. The preliminary investigations had not found the underlying genetic modifiers to support the double hit hypothesis, while the high level of estrogens in the female patients might exert a potential compensatory effect on mutant cell metabolism.

## CONFLICT OF INTEREST

The authors declare that they have no conflict of interest.
