# Supplementary material for: Bilateral striatal necrosis due to homoplasmic mitochondrial 3697G>A mutation presents with incomplete penetrance and sex bias
Source: Mol Genet Genomic Med. 2019 Jan 8;7(3):e541. doi: 10.1002/mgg3.541 (PMC6418351; doi:10.1002/mgg3.541)
Supplement: Supplementary file 1 [file MGG3-7-na-s001.pdf]

Supporting information:

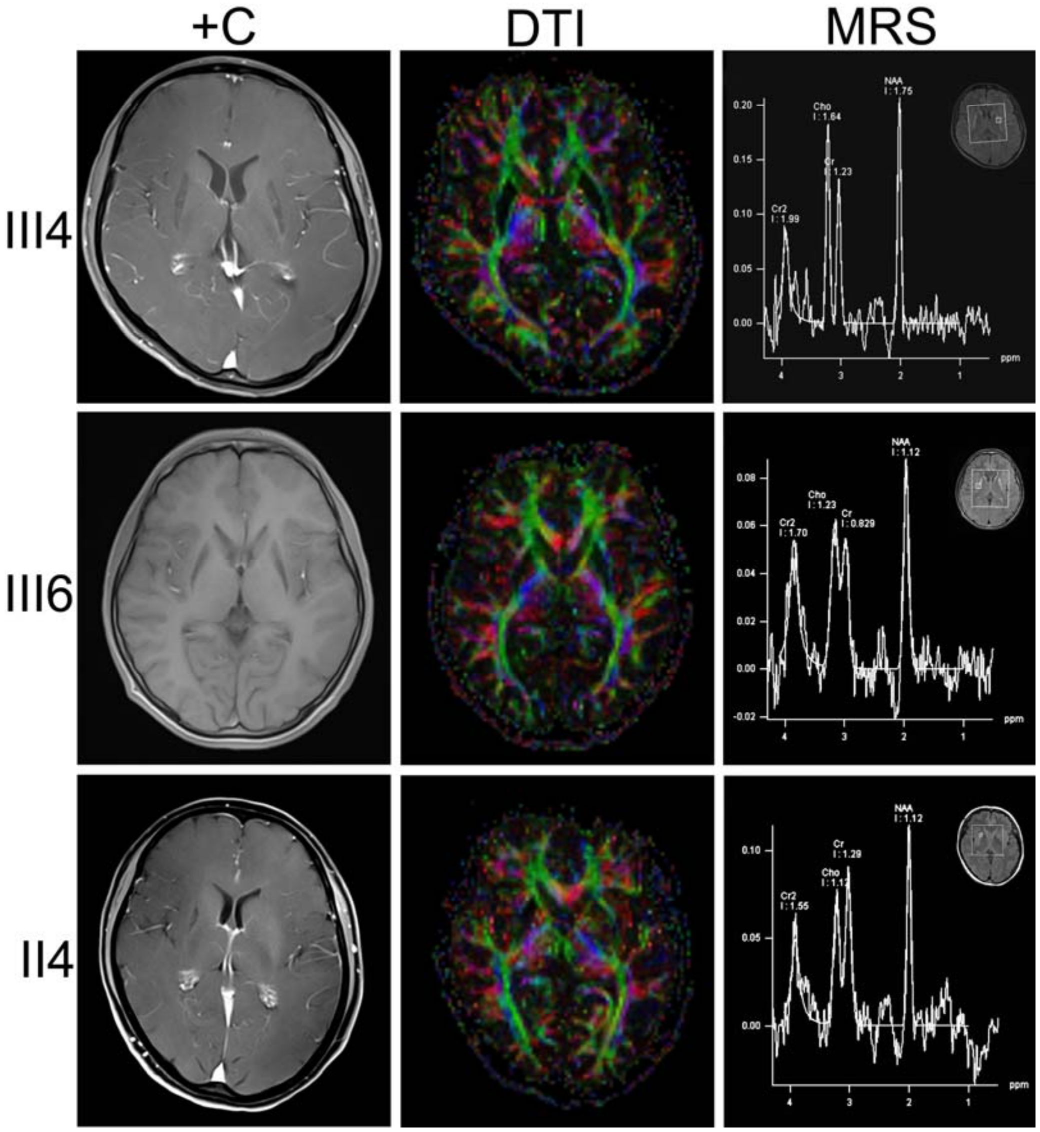

Figure S1. The striatal lesions were negative to enhancement, but the lesions showed the fiber interruption on diffusion tensor imaging (DTI), and severe decrease of n-acetylaspartate on magnetic resonance spectroscopy (MRS).

Table S1. mtDNA variants in the index patient with bilateral striatal necrosis.

**Gene**	**CRS Position**	**Replacement**	**Previously reported**
D-loop	73	A to G	Yes
	101	G to A	Yes
	150	C to T	Yes
	263	A to G	Yes
	315	C to CC	Yes
	456	C to T	Yes
	489	T to C	Yes
	16189	T to C	Yes
	16223	C to T	Yes
	16362	T to C	Yes
	16519	T to C	Yes
12s rRNA	681	T to C	Yes
	750	A to G	Yes
	1048	C to T	Yes
	1107	T to C	Yes
	1438	A to G	Yes
16s rRNA	2706	A to G	Yes
	3028	A to T	No
ND1	3697	G to A	Yes
	3759	A to G	Yes
ND2	4769	A to G	Yes
	4883	C to T	Yes
	5153	A to G	Yes
	5171	A to G	Yes
	5178	C to A	Yes
	5301	A to G	Yes
CO1	6253	T to C	Yes
	6286	T to T/C	Yes
	7028	C to T	Yes
ATP6	8701	A to G	Yes
	8860	A to G	Yes
	9180	A to G	Yes
	9540	T to C	Yes
ND3	10397	A to G	Yes
	10398	A to G	Yes
	10400	C to T	Yes
ND4	10873	T to C	Yes
	11719	G to A	Yes
ND5	12705	C to T	Yes
Cytb	14766	C to T	Yes
	14783	T to C	Yes
	15043	G to A	Yes
	15301	G to A	Yes
	15326	A to G	Yes
	15724	A to G	Yes

CRS, Cambridge reference sequence, See online mitochondrial genome databases: http://www.mitomap.org and http://www.genpat.uu.se/mtDB

Table S2. The list of candidate modifier genes for the gender bias.

Gene	Location	Variants	Patient	Sister	Amino acid change	Allele frequency	Function
Shroom2	Xp22.2	c.1426G>C	hemizygote	heterozygote	p.G476R	0.001796	amiloride-sensitive sodium channel
LUZP4	Xq23	c.382A>G	hemizygote	heterozygote	p.N128D	0.0002301	RNA export adapter

SHROOM2: shroom family member 2. LUZP4: leucine zipper protein 4.

Table S3. The summarization of clinical and laboratory data for patients with m.3697G>A mutation previously reported.

Literature	Case number	Gender	Age at onset	Clinical phenotype	MRI change	Mutation load of m.3697G>A	Biochemical results
Kirby et al.[1]	1	M	4y	hemiparesis MELAS	T2 high signal in the left basal ganglia and right frontal cortex	80% in muscle<br>79% in fibroblasts<br>3% in blood	complex I defect
Spruijt et al.[2]	2	F	35y	LHON	punctuated white matter T2 high signal	56% in blood	complex I defect
	3	M	3y	spastic dystonia, mental retardation, strabismus	T2 high signal in bilateral putamen.	88% in blood	complex I defect
Blakely et al.[3]	4	F	20y	migraine, deafness, MELAS, LHON	T2 high signal in occipital lobes and the thalami	98% in muscle<br>18% in blood<br>67% in urine	NA
Morava et al.[4]	5	M	neonatal	cardiomyopathy, muscle cramps, SWS	T2 high signal in the caudate nucleus	26% in muscle<br>20% in blood	decrease of pyruvate oxidation
	6	M	NA	encephalomyopathy	NA	80% in muscle<br>20% in blood	complex I defect
Negishi et al.[5]	7	F	1.5y	dystonia	T2 high signal in the bilateral putamen and caudate nucleus	homoplasmic	complex I defect
	8	M	1y	dystonia	T2 high signal in bilateral putamen, left globus pallidus, caudate nucleus and thalamus	homoplasmic	NA
	9	F	1y3m	dystonia and respiratory failure	T2 high signal in the bilateral putamen, caudate nucleus, globus pallidus and brainstem	homoplasmic	NA

MELAS: mitochondrial myopathy, encephalopathy, lactic acidosis and stroke-like episodes; LHON: Leber hereditary optic neuropathy; SWS: Stüve-Wiedemann

syndrome; NA: unavailable

Reference

1. Kirby DM, McFarland R, Ohtake A, Dunning C, Ryan MT, Wilson C, Ketteridge D, Turnbull DM, Thorburn DR, Taylor RW. Mutations of the mitochondrial ND1 gene as a cause of MELAS. J Med Genet. 2004 Oct;41(10):784-9. PubMed PMID: 15466014; PubMed Central PMCID: PMC1735602.
2. Spruijt L, Smeets HJ, Hendrickx A, Bettink-Remeijer MW, Maat-Kievit A, Schoonderwoerd KC, Sluiter W, de Coo IF, Hintzen RQ. A MELAS-associated ND1 mutation causing leber hereditary optic neuropathy and spastic dystonia. Arch Neurol. 2007 Jun;64(6):890-3. PubMed PMID: 17562939.
3. Blakely EL, de Silva R, King A, Schwarzer V, Harrower T, Dawidek G, Turnbull DM, Taylor RW. LHON/MELAS overlap syndrome associated with a mitochondrial MTND1 gene mutation. Eur J Hum Genet. 2005 May;13(5):623-7. PubMed PMID: 15657614.
4. Morava E, Hamel B, Hol F, Rodenburg R, Smeitink J. Mitochondrial dysfunction in Stüve-Wiedemann syndrome in a patient carrying an ND1 gene mutation. Am J Med Genet A. 2006 Oct 15;140(20):2248-50. PubMed PMID: 16969869.
5. Negishi Y, Hattori A, Takeshita E, Sakai C, Ando N, Ito T, Goto Y, Saitoh S. Homoplasmy of a mitochondrial 3697G>A mutation causes Leigh syndrome. J Hum Genet. 2014 Jul;59(7):405-7. doi: 10.1038/jhg.2014.41. Epub 2014 May 15. PubMed PMID: 24830958.
